# A Constitutive Description for Shape Memory Alloys with the Growth of Martensite Band

**DOI:** 10.3390/ma7010576

**Published:** 2014-01-20

**Authors:** Weiguo Li, Xueliang Shen, Xianghe Peng

**Affiliations:** State Key Laboratory of Coal Mine Disaster Dynamics and Control, College of Resources and Environment Science, Chongqing University, Chongqing 400030, China; E-Mails: xlshen@cqu.edu.cn (X.S.); xhpeng@cqu.edu.cn (X.P.)

**Keywords:** shape memory alloy, pseudoelasticity, volumetric strain, laminar microstructure, constitutive model

## Abstract

Based on the experimental results and the finite element analysis, a constitutive model is proposed for two phase shape memory alloys by introducing a compensative volumetric strain into a constrained relationship between the two phases, accounting for the reduced constraint due to the growth of martensite band. The pseudoelasticity of NiTi shape memory alloy micro-tube, subjected to pure tension, is analyzed and compared with the experimental results. It can be seen that the pseudoelastic behavior, especially the phenomena of a stress drop during tension processes, can be well described with the proposed model. The proposed model separates the complicated constitutive behavior of a shape memory alloy (SMA) into simple responses arising respectively from its two phases, taking into account laminar microstructure, the thickness of martensite phase and the interaction between the two phases, and provides an easy but comprehensive method for the description of the constitutive behavior of SMAs under complex thermomechanical loading.

## 1. Introduction

Shape memory alloys (SMAs) have been receiving increasing attention in recent years, due to their particular properties under thermomechanical loading, such as ferroelasticity, shape memory effect and pseudoelasticity. These properties are related to the martensitic phase transformation and are extensively used in many fields, such as aviation, national defense, instruments and medical devices, *etc.* The rapidly increasing applications of shape memory alloys require better understanding and more accurate description of their thermomechanical behavior, especially their behavior under complex multi-dimensional thermomechanical loading histories.

With the improvement of experimental facilities, many new experimental phenomena have been discovered [1,2]. Sun *et al.* [2] reported spiral band morphology on the surface of an SMA micro-tube subjected to tensile loading, and its nucleation and propagation with the progress of deformation. It was also reported that there is significant difference in the pseudoelastic behavior as well as the microstructures of the SMA micro-tube under pure torsion and under pure tension. Especially, a distinct stress drop appears as the macroscopic martensitic band grows during a tensile process.

It is recognized that the macroscopic property of a material strongly depends on its microstructures. Great progress has been made in the constitutive relationships for SMAs in the past 20 years, and especially in recent years [3–10]. However, the above significant behavior has not been successfully described, and the physical origin and quantitative model for the stress drop have not been well documented.

In this paper, the response of a NiTi SMA micro-tube subjected to pure tension is systematically investigated, taking into account the phase-transformation microstructures and their evolution. It is found that the physical and mechanical mechanism of the distinct stress drop can be attributed to the variation of the microstructure, *i.e*., the growth of the martensite band that strongly reduces the constraint between phases. The constitutive model is proposed based on the concept that a shape memory alloy is the mixture of martensite and austenite with laminated microstructure, taking the thickness of the martensitic band as an important parameter. The corresponding form of the constitutive model is obtained using the compensative volumetric strain, and the constitutive behavior of a NiTi SMA is analyzed and compared with experimental results. It can be seen that the pseudoelastic behavior, especially the phenomenon of stress drop during tension processes, can be well described with the proposed model.

## 2. Results and Discussion

A two-phase constitutive model for SMAs is proposed based on the concept that an SMA is composed of austenite and martensite, and the constitutive behavior of the SMA is substantially a combination of that of each phase. With the specified ranges of stress and temperature, the behavior of martensite is assumed elastoplastic while that of austenite is assumed linearly elastic. In the following part, the discussion is restricted to the case of small deformation and the material is assumed plastically incompressible.

### 2.1. A Constitutive Model for Martensitic Phase

**Figure 1.**
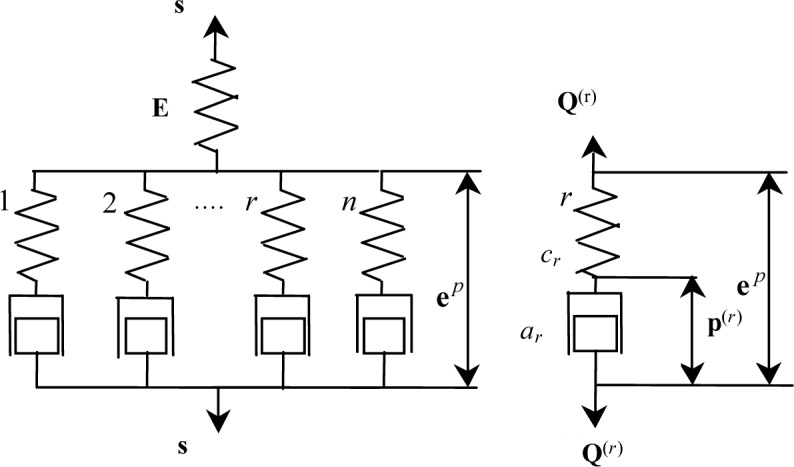
Mechanical model for the contribution of martensite.

Iwan (1967) [11] used a simple mechanical model consisting of elastic and plastic elements to describe the elastoplastic behavior of materials. It was extended to the model shown in Figure 1 for the description of the elastoplastic behavior of martensite. It contains a series of parallel Maxwell-type elements and an additional spring in series.

In Figure 1, a spring E is introduced to describe macroscopically elastic behavior of martensite. The *r*th dissipation mechanism is described by a spring *c_r_* and a dashpot-like block *a_r_*. *c_r_* is related to the stochastic internal structures and the energy stored in *c_r_* corresponds to that stored in the microstress fields induced by the respective pattern of defects at the microlevel. **e***^p^* denotes plastic strain component, and **Q**^(^*^r^*^)^ is the generalized force conjugated with the *r*th internal variable **p**^(^*^r^*^)^ and the following inequality should be satisfied if any change occurs to **p**^(^*^r^*^)^ [12,13].

$$\begin{array}{cc} Q^{\left(r\right)}:dp^{\left(r\right)}\geq 0 & \left(r=1,2,\cdots \cdots ,n\right) \end{array}$$ (1)

From Figure 1, it can be obtained that:

$$s=\overset{r=1}{\underset{n}{\Sigma }}Q^{\left(r\right)}$$ (2)

in which **Q**^(^*^r^*^)^ is assumed to relate the response of the *c_r_* and the flow of *a_r_* with the following phenomenological relationships:

$$Q^{\left(r\right)}=c_{r}\left(e^{p}-p^{\left(r\right)}\right)$$ (3)

$$Q^{\left(r\right)}=a_{r}\left(dp^{\left(r\right)}/dz\right)$$ (4)

where *z* is generalized time. It was observed by Sittner *et al.* [14] in biaxial tension-torsion tests that for some SMAs the response is not isotropic and the equivalent stress does not obey the von Mises rule. This phenomenon can be described by introducing the following defined generalized time:

$$\begin{array}{cc} dz=\frac{d\xi }{f(z)}, & {\left(d\xi \right)}^{2}=de^{p}:P:de^{p} \end{array}$$ (5)

where **P** is a material-dependent tensor of rank four and *f*(*z*) describes the change of the plastic damping property of the dashpot-like blocks *a_r_* (see Figure 1). It is assumed that:

$$C_{r}=C_{r}^{0}\varphi (T),\;a_{r}=a_{r}^{0}\varphi (T)$$ (6)

in which *T* denotes temperature.

The combination of Equations (3), (4) and (6) yields:

$$\Delta Q^{\left(r\right)}=K_{r}\varphi \left(T\right)C_{r}^{0}\Delta e^{p}-K_{r}\alpha_{r}Q^{\left(r\right)}\left({\bar{z}}_{n}\right)\Delta \bar{z}$$ (7)

where:

$$K_{r}=-\frac{e^{-\alpha_{r}\Delta \bar{z}}-1}{\alpha_{r}\Delta \bar{z}},\;{\text{α}}_{r}=\frac{C_{r}}{a_{r}},\;\Delta \bar{z}=\Delta z-\frac{d\phi }{\phi \cdot \alpha_{r}}$$ (8)

substituting Equation (7) into the differentiated form of Equation (2) yields:

$$K_{r}=-\frac{e^{-\alpha_{r}\Delta \bar{z}}-1}{\alpha_{r}\Delta \bar{z}},\;\Delta s=\sum_{r=1}^{n}\Delta Q^{\left(r\right)}=A\Delta e^{p}+B\left({\bar{z}}_{n}\right)\Delta z+\frac{\Delta \phi }{\phi }\bar{s}\left({\bar{z}}_{n}\right),\;\Delta \bar{z}=\Delta z-\frac{d\varphi }{\varphi \alpha_{r}}$$ (9)

in which:

$$A=\sum_{r=1}^{n}K_{r}C_{r},\;B=-\sum_{r=1}^{n}K_{r}\alpha_{r}Q^{\left(r\right)}\left({\bar{z}}_{n}\right),\;\bar{s}\left({\bar{z}}_{n}\right)=\sum_{r=1}^{n}K_{r}Q^{\left(r\right)}\left({\bar{z}}_{n}\right)$$ (10)

The deviatoric strain **e** can, in general, be assumed to consist of elastic component **e***^e^*, plastic component **e***^p^* and temperature induced recovery of inelastic distortion **e***^T^*, *i.e*.:

$$e=e^{e}+e^{p}+e^{T}$$ (11)

Making use of Equation (11), the elastic deviatoric response of the martensite phase can be expressed as:

$$s=2G\left(T\right)\left(e-e^{p}-e^{T}\right),\;\Delta s=\frac{\Delta G\left(T\right)}{G\left(T\right)}s+2G\left(T\right)\left(\Delta e-\Delta e^{p}-\Delta e^{T}\right)$$ (12)

For simplicity, the transformation lattice volume change is neglected in the present stage since for most SMAs it is negligible compared with the lattice shear deformation [15]. So, the volumetric stress of the martensite phase can be determined with:

$$tr\left(\sigma \right)=3K\left[tr\left(\varepsilon \right)-3\alpha \left(T-T_{0}\right)\right]$$ (13)

The differential of Equation (13) is:

$$tr\left(\Delta \sigma \right)=\frac{\Delta K}{K}tr\left(\sigma \right)+3K\left[tr\left(\Delta \varepsilon \right)-3\Delta \alpha \left(T-T_{0}\right)-3\alpha \Delta T\right]$$ (14)

Combining Equations (9), (12) and (14), one obtains:

$$\Delta s\;=\left[\frac{2GA}{A+2G}I_{4}+\frac{1}{a}B(z_{n})⊗\frac{{\left(2G\right)}^{2}\Delta e^{p}}{{\left(A+2G\right)}^{2}f^{2}\Delta z}\right]:\Delta e+\Delta \Psi$$ (15)

where:

$$\begin{array}{c} \Delta \Psi =\left[\frac{A}{A+2G}I_{4}+\frac{1}{a}B(z_{n})⊗\frac{2G\Delta e^{p}}{{\left(A+2G\right)}^{2}f^{2}\Delta z}\right]:\left[-2G(T)\Delta e^{T}+\frac{G'(T)}{G(T)}s\Delta T\right]+\\ \frac{\Delta \phi }{\phi }\frac{2G}{A+2G}\left[I_{4}-\frac{1}{a}B(z_{n})⊗\frac{\Delta e^{p}}{(A+2G)f^{2}\Delta z}\right]:\bar{s}\\ a=1+\frac{B:\Delta e^{p}}{(A+2G)f^{2}\Delta z} \end{array}$$ (16)

Keeping in mind that:

$$\Delta e^{p}:\Delta e=\Delta e^{p}:(\Delta \varepsilon -\frac{1}{3}\Delta \varepsilon_{kk}I_{2})=\Delta e^{p}:\Delta \varepsilon -\frac{1}{3}\Delta \varepsilon_{kk}\Delta e^{p}:I_{2}=\Delta e^{p}:\Delta \varepsilon$$ (17)

and:

$$\Delta s=\Delta \sigma -\frac{1}{3}tr(\Delta \sigma )I_{2},\;\Delta e=\Delta \varepsilon -\frac{1}{3}tr(\Delta \varepsilon )I_{2}$$ (18)

one can derive the following equation from Equation (15):

$$\Delta \sigma =\frac{A}{1+A/(2G)}(\Delta \varepsilon -\frac{1}{3}\Delta \varepsilon_{kk}I_{2})+\frac{{\left(2G\right)}^{2}}{{\left(2G+A\right)}^{2}f^{2}(z)}\frac{1}{a}B⊗\frac{\Delta e^{p}}{\Delta z}:\Delta \varepsilon +\Delta \Psi +\frac{1}{3}\Delta \sigma_{kk}I_{2}$$ (19)

Equation (19) can be written in the following matrix form by neglecting the terms containing ∆*T*. Assuming that the change of the temperature throughout this paper is not taken into account, *i.e*., it is an isothermal assumption:

{Δσ}=[D]{Δε} (20)

where:

$$D\;=\left[2G_{p}I_{4}+\left\{K-\frac{2}{3}G_{p}\right\}I_{2}⊗I_{2}+\frac{{\left(2G\right)}^{2}}{{\left(2G+A\right)}^{2}f^{2}(z)}\frac{1}{a}B⊗\frac{\Delta e^{p}}{\Delta z}+\Delta \Psi ⊗\frac{\Delta \varepsilon }{\Delta \varepsilon :\Delta \varepsilon }\right]$$ (21)

$$T_{P}={\left(1+\frac{A}{2G}\right)}^{-1},\;2G_{p}=AT_{P},\;\Delta z=\frac{\Delta e^{p}}{f^{2}\Delta z}:\Delta e^{p}$$ (22)

### 2.2. A Constitutive Model of Strain Compatibility Based on Volumetric Strain Compensation Method

**Figure 2.**
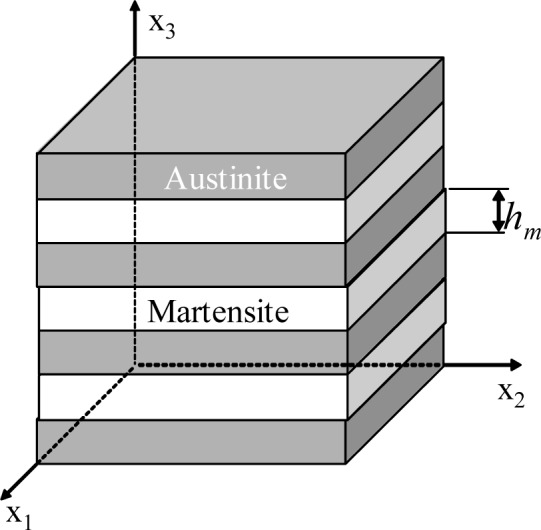
A representative volume element of SMA, *h_m_* is the thickness of martensite lamella.

In this research, the constitutive model is proposed for SMAs based on experimental results and finite element analysis, taking into account laminar microstructure, the thickness of martensite phase and the interaction between the two phases. The representative volume element (RVE) of an SMA is shown in Figure 2.

Assuming that the in-plane strain components and out-of-plane stress components in both martensite and austenite in all lamellae are identical and equal respectively to the corresponding components of the overall strain and stress in the SMA, the conventional mixture theory gives:

$$\Delta \varepsilon_{11}^{A}=\Delta \varepsilon_{11}^{M}=\Delta \varepsilon_{11},\;\Delta \varepsilon_{22}^{A}=\Delta \varepsilon_{22}^{M}=\Delta \varepsilon_{22},\;\Delta \varepsilon_{12}^{A}=\Delta \varepsilon_{12}^{M}=\Delta \varepsilon_{12}$$ (23)

$$\Delta \sigma_{33}^{A}=\Delta \sigma_{33}^{M}=\Delta \sigma_{33},\;\;\Delta \sigma_{23}^{A}=\Delta \sigma_{23}^{M}=\Delta \sigma_{23},\;\;\Delta \sigma_{13}^{A}=\Delta \sigma_{13}^{M}=\Delta \sigma_{13}$$ (24)

where the superscripts *A* and *M* represent austenite and martensite, respectively.

The other components of stress and strain can be determined by volume average as follows:

$$\sigma_{ij}=\xi \sigma_{ij}^{M}+(1-\xi )\sigma_{ij}^{A},\;ij=11,22,12$$ (25)

$$\varepsilon_{ij}=\xi \varepsilon_{ij}^{M}+(1-\xi )\varepsilon_{ij}^{A},\;ij=33,23,13$$ (26)

where ξ is the volume fraction of martensite.

The differential form of Equations (25) and (26) can be expressed as:

$$\left\{\begin{array}{c} \Delta \bar{\sigma }\\ \Delta \bar{\varepsilon } \end{array}\right\}=\xi \left\{\begin{array}{c} \Delta {\bar{\sigma }}_{M}\\ \Delta {\bar{\varepsilon }}_{M} \end{array}\right\}+\left(1-\xi \right)\left\{\begin{array}{c} \Delta {\bar{\sigma }}_{A}\\ \Delta {\bar{\varepsilon }}_{A} \end{array}\right\}+\Delta \xi \left\{\begin{array}{c} {\bar{\sigma }}_{M}-{\bar{\sigma }}_{A}\\ {\bar{\varepsilon }}_{M}-{\bar{\varepsilon }}_{A} \end{array}\right\}$$ (27)

with:

$$\begin{array}{l} \{\Delta \bar{\sigma }\}=(\Delta \sigma_{11},\;\Delta \sigma_{22},\;\Delta \tau_{12}){\;}^{T},\;\{\Delta \overset{⌢}{\sigma }\}=(\Delta \sigma_{33},\;\Delta \tau_{23},\;\Delta \tau_{31}){\;}^{T}\\ \{\Delta \overset{⌢}{\varepsilon }\}=(\Delta \varepsilon_{11},\;\Delta \varepsilon_{22},\;\Delta \varepsilon_{12}){\;}^{T},\;\{\Delta \bar{\varepsilon }\}=(\Delta \varepsilon_{33},\;\Delta \varepsilon_{23},\;\Delta \varepsilon_{31}){\;}^{T} \end{array}$$ (28)

The constitutive model for martensitic phase Equation (20) can be rewritten as:

$$\left\{\begin{array}{c} \Delta {\bar{\sigma }}_{M}\\ \Delta {\overset{⌢}{\sigma }}_{M} \end{array}\right\}=\left[\begin{array}{cc} A_{1}^{M} & A_{2}^{M}\\ A_{3}^{M} & A_{4}^{M} \end{array}\right]\left\{\begin{array}{c} \Delta {\overset{⌢}{\varepsilon }}_{M}\\ \Delta {\bar{\varepsilon }}_{M} \end{array}\right\}$$ (29)

where:

$$\begin{array}{l} \left[A_{1}^{M}\right]=\left[\begin{array}{ccc} D_{1111}^{M} & D_{1122}^{M} & D_{1112}^{M}\\ D_{2211}^{M} & D_{2222}^{M} & D_{2212}^{M}\\ D_{1211}^{M} & D_{1222}^{M} & D_{1212}^{M} \end{array}\right],\;\left[A_{2}^{M}\right]=\left[\begin{array}{ccc} D_{1133}^{M} & D_{1123}^{M} & D_{1131}^{M}\\ D_{2233}^{M} & D_{2223}^{M} & D_{2231}^{M}\\ D_{1233}^{M} & D_{1223}^{M} & D_{1231}^{M} \end{array}\right]\\ \left[A_{3}^{M}\right]=\left[\begin{array}{ccc} D_{3311}^{M} & D_{3322}^{M} & D_{3312}^{M}\\ D_{2311}^{M} & D_{2322}^{M} & D_{2312}^{M}\\ D_{3111}^{M} & D_{3122}^{M} & D_{3112}^{M} \end{array}\right],\;\left[A_{4}^{M}\right]=\left[\begin{array}{ccc} D_{3333}^{M} & D_{3323}^{M} & D_{3331}^{M}\\ D_{2333}^{M} & D_{2323}^{M} & D_{2331}^{M}\\ D_{3133}^{M} & D_{3123}^{M} & D_{3131}^{M} \end{array}\right] \end{array}$$ (30)

in which $D_{ijkl}^{M}$ denotes element *ijkl* in the tangential elastoplastic matrix of martensite [**D**_*M*_].

By the same way, one can easily obtain the following matrix:

$$\begin{array}{l} \left[A_{1}^{A}\right]=\left[\begin{array}{ccc} D_{1111}^{A} & D_{1122}^{A} & D_{1112}^{A}\\ D_{2211}^{A} & D_{2222}^{A} & D_{2212}^{A}\\ D_{1211}^{A} & D_{1222}^{A} & D_{1212}^{A} \end{array}\right],\;\left[A_{2}^{A}\right]=\left[\begin{array}{ccc} D_{1133}^{A} & D_{1123}^{A} & D_{1131}^{A}\\ D_{2233}^{A} & D_{2223}^{A} & D_{2231}^{A}\\ D_{1233}^{A} & D_{1223}^{A} & D_{1231}^{A} \end{array}\right]\\ \left[A_{3}^{A}\right]=\left[\begin{array}{ccc} D_{3311}^{A} & D_{3322}^{A} & D_{3312}^{A}\\ D_{2311}^{A} & D_{2322}^{A} & D_{2312}^{A}\\ D_{3111}^{A} & D_{3122}^{A} & D_{3112}^{A} \end{array}\right],\;\left[A_{4}^{A}\right]=\left[\begin{array}{ccc} D_{3333}^{A} & D_{3323}^{A} & D_{3331}^{A}\\ D_{2333}^{A} & D_{2323}^{A} & D_{2331}^{A}\\ D_{3133}^{A} & D_{3123}^{A} & D_{3131}^{A} \end{array}\right] \end{array}$$ (31)

where $D_{ijkl}^{A}$ denotes element *ijkl* in the tangential elastic matrix of austenite [**D**_*A*_].

Based on the study of the mechanical behavior, each phase responds when the two phases that coexist differ from the individual mechanical behavior of each phase. Volumetric strain compensation method is proposed to observe and study the effect of structure on the response of the mechanical behavior of each phase, while the hypothetical relation of strain compatibility is used to propose the constitutive relationship which can take into account the laminar spacing. Under the assumptions of small deformation—where isotropy is initially incompressible plastic—the simple mechanical model as in Figure 1 can still be used to describe the individual response of each phase.

Considering the constrained relationship between the two phases, one can use the following method to introduce the different effect of the constraint on the properties of the two phases.

$$\begin{array}{l} tr(\sigma_{M})=3K_{M}\left[tr(\varepsilon_{M})-3\alpha_{M}(T-T_{0})-\kappa \right]\;(a)\\ tr(\sigma_{A})=3K_{A}\left[tr(\varepsilon_{A})-3\alpha_{A}(T-T_{0})+\kappa \right]\;(b) \end{array}$$ (32)

in which κ is additional volumetric strain caused by different laminar spacing. This is the key point in this paper, which is quite different between this model and others. Additional volumetric strain is used to introduce the constrained relationship, which would be much more convenient and simple compared with other strains, while another model introduces the constrained relationship by the assumption that the in-plane strain has the following relationship [16]:

$$\varepsilon_{11}^{A}{}^{\prime }=\eta_{11}\varepsilon_{11}^{M}{}^{\prime },\varepsilon_{22}^{A}{}^{\prime }=\eta_{22}\varepsilon_{22}^{M}{}^{\prime },\varepsilon_{12}^{A}{}^{\prime }=\eta_{12}\varepsilon_{12}^{M}{}^{\prime }$$

where η is a factor of incompatible strain.

For soft phase, when it is tensed in the direction perpendicular to the lamina, the cross section of the soft phase will shrink more than the hard one, while the model assumes that the in-plane strain components should be the same. Inhibiting the shrinkage of the soft phase is in fact increasing the volumetric tensile stress in the soft phase, and it approximately works only when laminar spacing is small. However, when the laminar spacing is large, the in-plane constraint will decrease a lot, so much as to disappear. When it is tensed in parallel to the direction of the lamina, soft phase advance into the plastic deformation as the Poisson ratio of the plastic deformation is larger, so there is also a concern that the volumetric shrinkage was constrained.

The differential of Equation (32a) is:

$$tr(\Delta \sigma )=\frac{\Delta K}{K}tr(\sigma )+3K\left[tr(\Delta \varepsilon )-3\Delta \alpha (T-T_{0})-3\alpha \Delta T-\Delta \kappa \right]$$ (33)

The parameter κ can be simply shown as:

$$\kappa =g(h)\left[tr(\varepsilon_{M})-tr(\varepsilon_{A})\right]$$ (34)

where *g*(*h*) is the parameter of between additional volumetric strain and the difference of the volumetric strain of the two phases.

The differential of Equation (34) is:

$$\Delta \kappa =\Delta g(h)\left[tr(\varepsilon_{M})-tr(\varepsilon_{A})\right]+g(h)\left[tr(\Delta \varepsilon_{M})-tr(\Delta \varepsilon_{A})\right]$$ (35)

It can be obtained from Equation (35) that:

$$\begin{array}{l} \Delta \sigma_{kk}=\frac{\Delta K}{K}\sigma_{kk}+3K\Delta \varepsilon_{kk}-9K\Delta \alpha (T-T_{0})-9K\alpha \Delta T-3K\Delta \kappa \\ \;\;=3K\Delta \varepsilon_{kk}+\left[\frac{K'}{K}\sigma_{kk}-9K\alpha '(T-T_{0})-9K\alpha \right]\Delta T-\\ \;\;3K\left[\Delta g(h)\left[tr(\varepsilon_{M})-tr(\varepsilon_{A})\right]+g(h)\left[tr(\Delta \varepsilon_{M})-tr(\Delta \varepsilon_{A})\right]\;\right] \end{array}$$ (36)

For martensitic phase, Equation (36) can be expressed as:

$$\Delta \sigma_{kk}=3K\left[1-g(h)\right]\Delta \varepsilon_{kk}+\Delta v_{M}$$ (37)

where:

$$\Delta v_{M}=\left[\frac{K'}{K}\sigma_{kk}-9K\alpha '(T-T_{0})-9K\alpha \right]\Delta T-3K\left[g'(h)\left(\varepsilon_{kk}-\varepsilon_{kk}^{A}\right)\Delta h-g\left(h\right)\Delta \varepsilon_{kk}^{A}\right]$$ (38)

For austentic phase, one can easily obtain the following equation from Equation (32b) by the same way:

$$\Delta \sigma_{kk}=3K\left[1-g(h)\right]\Delta \varepsilon_{kk}+\Delta v_{A}$$ (39)

where:

$$\Delta v_{A}=\left[\frac{K'}{K}\sigma_{kk}-9K\alpha '(T-T_{0})-9K\alpha \right]\Delta T-3K\left[g'(h)\left(\varepsilon_{kk}-\varepsilon_{kk}^{M}\right)\Delta h-g\left(h\right)\Delta \varepsilon_{kk}^{M}\right]$$ (40)

in which κ and α are respectively corresponding to martensitic and austenitic material parameters from Equations (37)–(40).

For martensite:

$$\begin{array}{l} \Delta \sigma =\frac{A}{1+A/(2G)}(\Delta \varepsilon -\frac{1}{3}\Delta \varepsilon_{kk}I_{2})+\frac{{\left(2G\right)}^{2}}{{\left(2G+A\right)}^{2}f^{2}(z)}\frac{1}{a}B⊗\frac{\Delta e^{p}}{\Delta z}:\Delta \varepsilon +\Delta \Psi +\frac{1}{3}\Delta \sigma_{kk}I_{2}\\ \;=\frac{A}{1+A/(2G)}(\Delta \varepsilon -\frac{1}{3}\Delta \varepsilon_{kk}I_{2})+\frac{{\left(2G\right)}^{2}}{{\left(2G+A\right)}^{2}f^{2}(z)}\frac{1}{a}B⊗\frac{\Delta e^{p}}{\Delta z}:\Delta \varepsilon +\Delta \Psi +\frac{1}{3}\left\{3K\left[1-g(h)\right]\Delta \varepsilon_{kk}+\Delta v_{M}\right\}I_{2}\\ \;=\left\{\left[\begin{array}{l} 2G_{p}I_{4}+\left\{K\left[1-g(h)\right]-\frac{2}{3}G_{p}\right\}I_{2}⊗I_{2}+\frac{{\left(2G\right)}^{2}}{{\left(2G+A\right)}^{2}f^{2}(z)}\frac{1}{a}B⊗\frac{\Delta e^{p}}{\Delta z}+\\ (\Delta \Psi +\Delta v_{M}I_{2})⊗\frac{\Delta \varepsilon }{\Delta \varepsilon :\Delta \varepsilon } \end{array}\right]\right\}:\Delta \varepsilon \end{array}$$ (41)

that is:

$$\left\{\Delta \sigma \right\}=\left[D_{M}\right]\left\{\Delta \varepsilon \right\}$$ (42)

in which:

$$D_{M}\;=\left[\begin{array}{l} 2G_{p}I_{4}+\left\{K\left[1-g(h)\right]-\frac{2}{3}G_{p}\right\}I_{2}⊗I_{2}+\frac{{\left(2G\right)}^{2}}{{\left(2G+A\right)}^{2}f^{2}(z)}\frac{1}{a}B⊗\frac{\Delta e^{p}}{\Delta z}+\\ (\Delta \Psi +\Delta v_{M}I_{2})⊗\frac{\Delta \varepsilon }{\Delta \varepsilon :\Delta \varepsilon } \end{array}\right]$$ (43)

By the same way, austenitic constitutive equation can be written as

$$\left\{\Delta \sigma \right\}=\left[D_{A}\right]\left\{\Delta \varepsilon \right\}$$ (44)

where:

$$D_{A}\;=\left[\begin{array}{l} 2G_{p}I_{4}+\left\{K\left[1+g(h)\right]-\frac{2}{3}G_{p}\right\}I_{2}⊗I_{2}+\frac{{\left(2G\right)}^{2}}{{\left(2G+A\right)}^{2}f^{2}(z)}\frac{1}{a}B⊗\frac{\Delta e^{p}}{\Delta z}+\\ (\Delta \Psi +\Delta v_{A}I_{2})⊗\frac{\Delta \varepsilon }{\Delta \varepsilon :\Delta \varepsilon } \end{array}\right]$$ (45)

*g*(*h*) in Equation (34) can be defined as:

$$h\leq 1/2:g(h)=\frac{b_{1}}{2}\left[1-\text{exp}(-d_{1}h)\right]$$ (46)

$$h\geq 1/2:g(h)=\frac{b_{2}}{2}\left[1-\text{exp}(-d_{2}(1-h))\right]$$ (47)

where *h* stands for relative thickness: *h* is equal to the thickness of martensite lamella/(the thickness of martensite lamella + the thickness of austenite lamella); it can be obtained that *h* is the volume fraction of martensite, which changes from 0 to 1. When *h* approaches 0 or 1, *g*(*h*) is close to 0. The value of *b*_1_ and *b*_2_ is decided by the difference of the material properties of the two phases. The value of *d*_1_ and *d*_2_ can be obtained from the finite element analysis, while *d*_1_ and *d*_2_ can contain information on laminar spacing so that it can introduce the effect of the laminar spacing. For SMA, in order to reflect that the effect of the hard and soft phase on incompatibility of two phases is different when their volumetric fraction change, generally speaking, there yields

$$d_{1}\left(h\right)\geq d_{2}(1-h)$$ (48)

when *h* = 1/2 : *d*_1_(h) = *d*_2_(1−h).

Taking into account the laminar microstructure, the dynamic change of the thickness of martensite and austenite during phase transformation and the constraints of the two phases, the in-plane strain coordinate and out-plane stress coordinate can be deduced, so that the constitutive relationships for SMAs which take into account the laminar microstructure can be obtained.

Equation (27) can be written as:

$$\begin{array}{l} \left\{\begin{array}{c} \Delta \bar{\sigma }\\ \Delta \bar{\varepsilon } \end{array}\right\}=\xi \left\{\begin{array}{c} \Delta {\bar{\sigma }}_{M}\\ \Delta {\bar{\varepsilon }}_{M} \end{array}\right\}+\left(1-\xi \right)\left\{\begin{array}{c} \Delta {\bar{\sigma }}_{A}\\ \Delta {\bar{\varepsilon }}_{A} \end{array}\right\}+\Delta \xi \left\{\begin{array}{c} {\bar{\sigma }}_{M}-{\bar{\sigma }}_{A}\\ {\bar{\varepsilon }}_{M}-{\bar{\varepsilon }}_{A} \end{array}\right\}\\ \begin{array}{ccc} & & =\left[\begin{array}{cc} B_{1} & B_{2}\\ B_{3} & B_{4} \end{array}\right]\left\{\begin{array}{c} \Delta \overset{⌢}{\sigma }\\ \Delta \overset{⌢}{\varepsilon } \end{array}\right\}+\Delta \xi \left\{\begin{array}{c} {\bar{\sigma }}_{M}-{\bar{\sigma }}_{A}\\ {\bar{\varepsilon }}_{M}-{\bar{\varepsilon }}_{A} \end{array}\right\} \end{array} \end{array}$$ (49)

It can be obtained from Equation (49) that:

$$\Delta \bar{\sigma }=B_{1}\Delta \overset{⌢}{\sigma }+B_{2}\Delta \overset{⌢}{\varepsilon }+\Delta \xi \left({\bar{\sigma }}_{M}-{\bar{\sigma }}_{A}\right)$$ (50)

$$\Delta \bar{\varepsilon }=B_{3}\Delta \overset{⌢}{\sigma }+B_{4}\Delta \overset{⌢}{\varepsilon }+\Delta \xi \left({\bar{\varepsilon }}_{M}-{\bar{\varepsilon }}_{A}\right)$$ (51)

It can also be obtained from Equation (51) that:

$$\Delta \overset{⌢}{\sigma }=B_{3}^{-1}\Delta \bar{\varepsilon }-B_{3}^{-1}B_{4}\Delta \overset{⌢}{\varepsilon }-B_{3}^{-1}\Delta \xi \left({\bar{\varepsilon }}_{M}-{\bar{\varepsilon }}_{A}\right)$$ (52)

Substituting Equation (52) into Equation (50) yields:

$$\begin{array}{l} \Delta \bar{\sigma }=B_{1}\left[B_{3}^{-1}\Delta \bar{\varepsilon }-B_{3}^{-1}B_{4}\Delta \overset{⌢}{\varepsilon }-B_{3}^{-1}\Delta \xi \left({\bar{\varepsilon }}_{M}-{\bar{\varepsilon }}_{A}\right)\right]+B_{2}\Delta \overset{⌢}{\varepsilon }+\Delta \xi \left({\bar{\sigma }}_{M}-{\bar{\sigma }}_{A}\right)\\ =B_{1}B_{3}^{-1}\Delta \bar{\varepsilon }-\left(B_{1}B_{3}^{-1}B_{4}-B_{2}\right)\Delta \overset{⌢}{\varepsilon }-B_{1}B_{3}^{-1}\Delta \xi \left({\bar{\varepsilon }}_{M}-{\bar{\varepsilon }}_{A}\right)+\Delta \xi \left({\bar{\sigma }}_{M}-{\bar{\sigma }}_{A}\right) \end{array}$$ (53)

Equations (52) and (53) can be written in the following matrix form:

$$\left\{\begin{array}{c} \Delta \bar{\sigma }\\ \Delta \overset{⌢}{\sigma } \end{array}\right\}=\left[\begin{array}{cc} B_{2}-B_{1}B_{3}^{-1}B_{4} & B_{1}B_{3}^{-1}\\ -B_{3}^{-1}B_{4} & B_{3}^{-1} \end{array}\right]\left\{\begin{array}{c} \Delta \overset{⌢}{\varepsilon }\\ \Delta \bar{\varepsilon } \end{array}\right\}+\Delta \xi \left[\begin{array}{c} -B_{1}B_{3}^{-1}\left({\bar{\varepsilon }}_{M}-{\bar{\varepsilon }}_{A}\right)+\left({\bar{\sigma }}_{M}-{\bar{\sigma }}_{A}\right)\\ -B_{3}^{-1}\left({\bar{\varepsilon }}_{M}-{\bar{\varepsilon }}_{A}\right) \end{array}\right]$$ (54)

From the response formula of the martensite phase Equation (42), one can obtain that:

$$\left\{\begin{array}{c} \Delta {\bar{\sigma }}_{M}\\ \Delta {\overset{⌢}{\sigma }}_{M} \end{array}\right\}=\left[\begin{array}{cc} A_{1}^{M} & A_{2}^{M}\\ A_{3}^{M} & A_{4}^{M} \end{array}\right]\left\{\begin{array}{c} \Delta {\overset{⌢}{\varepsilon }}_{M}\\ \Delta {\bar{\varepsilon }}_{M} \end{array}\right\}$$ (55)

Expanding Equation (55) yields:

$$\begin{array}{l} \begin{array}{cc} \Delta {\bar{\sigma }}_{M}=A_{1}^{M}\Delta {\overset{⌢}{\varepsilon }}_{M}+A_{2}^{M}\Delta {\bar{\varepsilon }}_{M} & \left(a\right) \end{array}\\ \begin{array}{cc} \Delta {\overset{⌢}{\sigma }}_{M}=A_{3}^{M}\Delta {\overset{⌢}{\varepsilon }}_{M}+A_{4}^{M}\Delta {\bar{\varepsilon }}_{M} & \left(b\right) \end{array} \end{array}$$ (56)

One can derive the following equation from Equation (56b):

$$\Delta {\bar{\varepsilon }}_{M}={\left[A_{4}^{M}\right]}^{-1}\Delta {\overset{⌢}{\sigma }}_{M}-{\left[A_{4}^{M}\right]}^{-1}A_{3}^{M}\Delta {\overset{⌢}{\varepsilon }}_{M}$$ (57)

Substituting Equation (57) into Equation (56a) yields:

$$\Delta {\bar{\sigma }}_{M}=\left(A_{1}^{M}-A_{2}^{M}{\left[A_{4}^{M}\right]}^{-1}A_{3}^{M}\right)\Delta {\overset{⌢}{\varepsilon }}_{M}+A_{2}^{M}{\left[A_{4}^{M}\right]}^{-1}\Delta {\overset{⌢}{\sigma }}_{M}$$ (58)

Equations (57) and (58) can be written in the following matrix form:

$$\left\{\begin{array}{c} \Delta {\bar{\sigma }}_{M}\\ \Delta {\bar{\varepsilon }}_{M} \end{array}\right\}=\left[\begin{array}{cc} A_{2}^{M}{\left[A_{4}^{M}\right]}^{-1} & A_{1}^{M}-A_{2}^{M}{\left[A_{4}^{M}\right]}^{-1}A_{3}^{M}\\ {\left[A_{4}^{M}\right]}^{-1} & -{\left[A_{4}^{M}\right]}^{-1}A_{3}^{M} \end{array}\right]\left\{\begin{array}{c} \Delta {\overset{⌢}{\sigma }}_{M}\\ \Delta {\overset{⌢}{\varepsilon }}_{M} \end{array}\right\}$$ (59)

Similarly, for austenite phase, one can easily obtain that:

$$\left\{\begin{array}{c} \Delta {\bar{\sigma }}_{A}\\ \Delta {\bar{\varepsilon }}_{A} \end{array}\right\}=\left[\begin{array}{cc} A_{2}^{A}{\left[A_{4}^{A}\right]}^{-1} & A_{1}^{A}-A_{2}^{A}{\left[A_{4}^{A}\right]}^{-1}A_{3}^{A}\\ {\left[A_{4}^{A}\right]}^{-1} & -{\left[A_{4}^{A}\right]}^{-1}A_{3}^{A} \end{array}\right]\left\{\begin{array}{c} \Delta {\overset{⌢}{\sigma }}_{A}\\ \Delta {\overset{⌢}{\varepsilon }}_{A} \end{array}\right\}$$ (60)

Substituting Equations (59) and (60) into Equation (49) yields:

$$\begin{array}{l} B_{1}=\xi A_{2}^{M}{\left[A_{4}^{M}\right]}^{-1}+(1-\xi )A_{2}^{A}{\left[A_{4}^{A}\right]}^{-1}\\ B_{2}=\xi \left[A_{1}^{M}-A_{2}^{M}{\left[A_{4}^{M}\right]}^{-1}A_{3}^{M}\right]+(1-\xi )\left[A_{1}^{A}-A_{2}^{A}{\left[A_{4}^{A}\right]}^{-1}A_{3}^{A}\right]\\ B_{3}=\xi {\left[A_{4}^{M}\right]}^{-1}+(1-\xi ){\left[A_{4}^{A}\right]}^{-1}\\ B_{4}=-\xi {\left[A_{4}^{M}\right]}^{-1}A_{3}^{M}-(1-\xi ){\left[A_{4}^{A}\right]}^{-1}A_{3}^{A} \end{array}$$ (61)

Among them: the concrete forms of $\left[A_{1}^{M}\right]$, $\left[A_{2}^{M}\right]$, $\left[A_{3}^{M}\right]$, $\left[A_{4}^{M}\right]$, $\left[A_{1}^{A}\right]$, $\left[A_{2}^{A}\right]$, $\left[A_{3}^{A}\right]$, $\left[A_{4}^{A}\right]$ are respectively consistent with Equations (30) and (31), $D_{ijkl}^{M}$, $D_{ijkl}^{A}$ in the equations are respectively corresponding to elements of elastoplastic matrix [**D**_*M*_] of martensite in Equation (43) and elastic matrix [**D**_*A*_] of austenite in Equation (45). Equation (54) is the constitutive relationship of the SMA considering laminar microstructure, this constitutive relationship considers the effect of the laminar spacing.

## 3. Experimental Section

Sun *et al.* [2] made a detailed investigation on the behavior of pseudoelastic NiTi (49–51Ni) SMA micro-tubes under tension and torsion. The main constitutive behavior of the material will be described with the proposed constitutive model.

Since the experiment was performed at room temperature and under quasistatic condition, the effect of temperature on the material properties can be neglected. In Equation (2) and Equation (9), *n = 3* is selected to satisfy the requirement of both the accuracy and efficiency in the analysis for practical engineering problems [17]. For the sake of simplicity without losing generality, *f_M_* *=* exp(*h*ε*_ij_*) for martensite phase because of reorientations of martensite variants, where ε*_e_* = (2ε*_ij_*ε*_ij_*/3)^1/2^ is equivalent strain, and *h* = 4.908. For the description of uniaxial constitutive behavior, P can be taken as an identity tensor. The other material constants were identified as follows:

The values of the material constants *c_r_* and *a_r_* are obtained by experiment as in Figure 1. When it can simulate the single respond of the martensite (austenite), the values of the material constants c_r_ and a_r_ are fixed. So, the material constants *c_r_* and *a_r_* are given in this paper.

**Figure 3.**
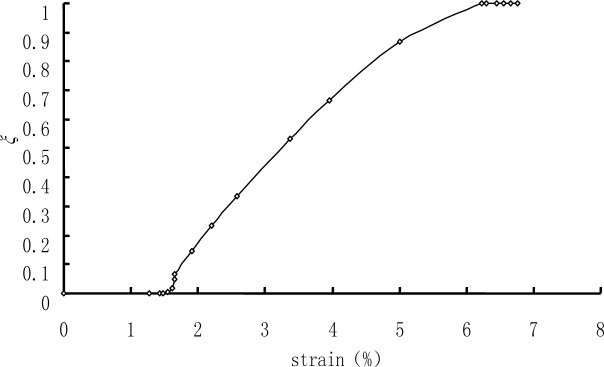
The variation of tension–strain *versus* martensite volume fraction (see Li, 2005 [18]). ξ stands for the volume fraction of martensite under uniaxial tension. The volume fraction of martensite is obtained from the finite element analysis when the strain varies from 0%–1.6%; elsewhere it is obtained by experimental observation.

The numerical process is strain-controlled. For uniaxial tension—keeping in mind that the nucleation and propagation of a macroscopic martensite band was observed during the test under uniaxial tension, so its microstructure is simplified to be laminar—the relationship of the volume fraction of martensite ξ and the stretched strain is showed by Figure 3 based on the experimental results, and the actual relationship of the in-plane strain components in both martensite and austenite are assumed to obey the relations which were obtained by finite element analysis [18].

**Figure 4.**
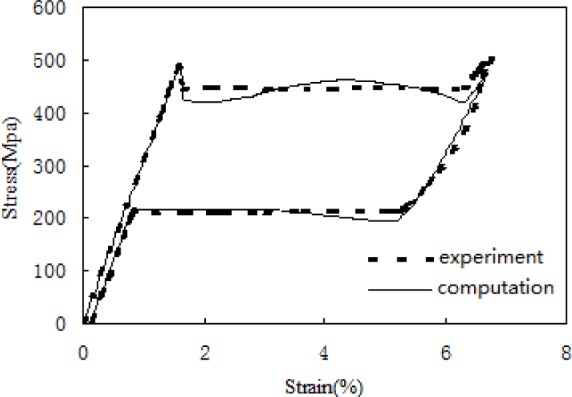
The nominal tensile stress–strain curve of micro-tube.

**Figure 5.**
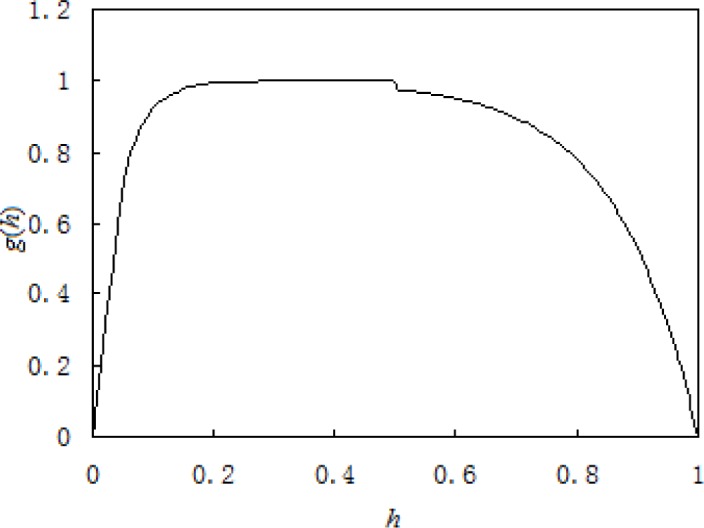
The relationship between *h* and *g*(*h*) which is given in Equations (46) and (47). *h* is equal to the thickness of martensite lamella/(the thickness of martensite lamella + the thickness of austenite lamella), which stands for relative thickness.

Using the constitutive model of strain compatibility based on volumetric compensation which is obtained in the previous chapter to compute mechanical behavior of a micro-tube under pure tensile: in the concrete computation *b*_1_ = *b*_2_ = 2, *d*_1_ = 25, *d*_2_ = 7.5 (material constants are listed in Table 1), it can be seen from Figure 4 that the behavior of a micro-tube under pure tensile is well described with the proposed model. Especially, the typical stress drop during tension processes can be well described with the proposed microstructure-based model. However, in the concrete computation, as the functions *d*_1_ and *d*_2_ have been assigned fixed values, it makes the computation locally different with the results of the experiment, and at the same time, it also makes *g*(*h*) in Figure 5 slightly jump when *h* = 1/2. So, future work should be done to acquire the concrete functions *d*_1_ and *d*_2_ by finite element analysis in order to be better used for the computation of the proposed constitutive model.

## 4. Conclusions

A two-phase constitutive model for polycrystalline SMAs with laminar microstructure—based on the concept that a SMA is composed of austenite and martensite—and a new way to introduce the constraints of the two phases are proposed. The main characteristics of SMAs such as ferroelasticity and pseudoelasticity can be described with the proposed model and especially the phenomena of a stress drop during tension processes. This constitutive model can also explain the stress–strain relationship of other complicated structural materials like functionally graded material [19] and laminated composite [20].

The responses of a NiTi SMA micro-tube subjected to pure tension were investigated and compared with the experimental results, taking into account the phase-transformation microstructures. The comparison between the calculated and experimental results shows satisfactory agreement.

The complicated constitutive behavior of a polycrystalline SMA is separated into the simple constitutive behavior of its two phases under the constraint with each other, which provides a simple but comprehensive description for the constitutive behavior of SMAs. The ratio of the in-plane strain components between martensite to austenite η is an important parameter in the model, which is the function of the stress state σ, temperature *T*, the volume fraction of martensite ξ and the thickness of martensite band, so that the laminar microstructure, thickness of martensite phase, interaction between the two phases and their evolution can be easily taken into account.

## Figures and Tables

**Table 1.** Material constants.

Material	G (GPa)	*ν*	*c*_1_; *c*_2_; *c*_3_ (GPa)	α_1_; α_2_; α_3_
Martensite	18.45	0.167	50,000; 0; 0.	500; 60; 5
Austenite	13.8	0.167	16,500,000; 500,000; 0.	30,000; 2000; 500
